# Wavelength dependence of fine spatial resolution in human vision

**DOI:** 10.3758/s13414-026-03244-5

**Published:** 2026-05-29

**Authors:** Yaw Buabeng, Billy R. Hammond

**Affiliations:** Vision Sciences Laboratory, Behavioral and Brain Sciences Program, University of Georgia, Athens, GA 30602 USA

**Keywords:** Light scatter, Wavelengths, Iris color, Visual function, Spatial resolution

## Abstract

Wavelength influences multiple aspects of visual performance, yet its role in spatial resolution remains incompletely understood due to confounding factors such as luminance differences, chromatic aberration, and intraocular scatter. This study assessed how narrowband light of different wavelengths affects two-point separation thresholds under controlled stimulus and ocular conditions. Action spectra for fine spatial resolution were measured using an equal-energy approach. Sixty healthy young adults (mean age: 22.7 ± 3.3 years) with normal vision were tested in a two-point resolution task. Narrowband stimuli (420–660 nm) and a broadband white condition were produced by a 1,000-W Xenon arc lamp with interference filters. Participants were preselected for optimal acuity. Thresholds, defined as the minimum resolvable separation between two-point sources (two-point separation thresholds), were recorded using a digital micrometer, and converted to visual angle for analysis. Separation thresholds varied significantly with wavelength with short-wave light (420 nm) yielding poorer resolution compared to long-wave light (660 nm). Iris pigmentation (color, lightness, and a combination of color + lightness) also influenced performance with lighter irides associated with higher thresholds, and the largest group differences observed at short wavelengths. Two-point resolution shows systematic wavelength dependence under equal-energy conditions, with performance degraded in the short-wave range. These effects likely reflect the combined influence of chromatic aberration, optical scatter, and photoreceptor sampling, rather than scatter alone. Consideration of both optical and neural mechanisms is essential when interpreting wavelength-dependent changes in spatial vision.

## Introduction

Action spectroscopy is broadly defined as the measurement of biological effects as a function of wavelength (Coohill, 1991). Its origins trace back to plant physiology, where wavelength-dependent responses led to the identification of chlorophyll as the primary pigment in photosynthesis (Charles Giles Bridle Daubeny, 1836; McCree, 1971). The same principles were later generalized to animal photobiology, first emphasizing damage from specific spectral bands (Hockberger, 2007), and subsequently extending to visual and non-visual photoreception.

In vision science, action spectra have long provided fundamental insights into photoreceptor sensitivity and visual performance. Early human spectral sensitivity curves (CIE, 1932) spurred decades of work examining wavelength-dependent effects on temporal (de Lange Dzn, 1958), spatial (Campbell & Green, 1965), and discomfort responses (Flannagan et al., 1990). For example, glare discomfort has been shown to scale disproportionately with short-wave light, whether assessed by squint response or self-report (Stringham et al., 2003).

A central challenge in such studies is stimulus control**.** Because the human eye’s spectral sensitivity varies strongly across wavelengths, results can be confounded if stimuli are equated by radiometric (energy-based) rather than photometric (luminance-based) measures (or vice versa). This distinction is critical: radiometric matching allows examination of intrinsic wavelength effects, while photometric matching incorporates brightness perception but obscures the direct impact of wavelength (Pokorny et al., 2003; Wyszecki & Stiles, 2000). Photometric matching would necessarily require substantially greater short-wave radiant energy to achieve equal luminance, thereby altering retinal illuminance, adaptation state, and the very optical mechanisms (e.g., chromatic aberration and straylight) that an action-spectrum approach seeks to isolate. Relatedly, other uncontrolled factors such as pupil size, accommodation, refractive error, and higher-order aberrations, can substantially influence spatial resolution yet are rarely accounted for (Campbell & Gregory, 1960; Thibos et al., 1992).

One factor of particular interest is intraocular light scatter**.** While Rayleigh and Mie theory predicts wavelength dependence in atmospheric optics, intraocular scattering is less straightforward. Some reports suggest little or no wavelength effect (Ginis et al., 2012; Wooten & Geri, 1987), while others demonstrate greater short-wave scatter consistent with Rayleigh predictions (Coppens et al., 2006; Whitaker et al., 1993). Discrepancies may reflect differences in methodology, ocular pigmentation, or age-related media changes.

Although the influence of wavelength on spectral sensitivity has been extensively characterized, far less is known about how wavelength impacts spatial discrimination thresholds. Two-point resolution is a fundamental measure of visual performance, shaped by both optical factors (diffraction, scatter, chromatic aberration) and neural processing limits. Understanding how resolution varies across the visible spectrum provides insight into the perceptual consequences of wavelength-dependent optics and the interaction between optical and neural constraints. The present study therefore examined action spectra for fine spatial resolution using a controlled psychophysical paradigm. By quantifying how thresholds change as a function of wavelength and iris pigmentation, this work contributes to a broader understanding of how optical properties of the eye shape basic perceptual limits.

## Methods

### Study design and population

Sixty healthy young adults (60% female, 40% male; mean age = 22.73 years, *SD* = 3.33) participated. Descriptive statistics are reported in Table 1. Ocular history was obtained via self-report. Inclusion criteria were age 18–30 years of ageand uncorrected visual acuity better than 20/40 in each eye, measured using a Snellen acuity chart. All participants reported normal color vision. Visual acuity was tested without habitual correction, and the dominant eye was identified prior to testing. To avoid confounding effects of optical corrections, no subject wore glasses or contact lenses during the experiment. Exclusion criteria included ocular conditions that could directly interfere with optical quality or light scatter (e.g., corneal infection, stye, or other anterior segment abnormalities).

**Table 1:** Demographic characteristics of the participants (*n* = 60)

Variable	Categories	Percent of sample
Race	Black/African American	31.7
	White/Caucasian	51.7
	Asian/Pacific Islander	13.3
	Latino/a	3.3
Ethnicity	Hispanic	3.3
	Non-Hispanic	96.7
Gender	Woman	60
	Man	40
	Other/Third	0
Iris Lightness	Light	11.7
	Medium	43.3
	Dark	45.0
Iris Color	Blue	15.0
	Green	11.7
	Hazel	11.7
	Brown	61.7

Iris color was assessed by visual inspection under standardized indoor illumination and categorized according to the photographic reference system of Mackey et al. (2011). Although the Mackey system has nine categories, the participants were (coincidentally) described by just four of the color groupings (blue, green, hazel, brown). Independently of color, irides were also classified by overall pigment density into light, medium, and dark categories. This lightness classification was based on visible stromal pigment density under standardized illumination, corresponding approximately to lower (light), intermediate (medium), and higher (dark) pigment groupings in the Mackey et al. reference set. Because iris color clustered empirically into three lightness categories, these groupings were used in subsequent analyses. The classifications were performed prior to data analysis and without reference to the behavioral outcomes. Participants were recruited from the undergraduate and graduate student population at the University of Georgia. The study protocol was approved by the University of Georgia Institutional Review Board (PROJECT00009383). Procedures followed Good Clinical Practice Guidelines and the tenets of the Declaration of Helsinki.

### Apparatus and stimuli

A detailed schematic of the apparatus can be found in Buabeng and Hammond (2025). A 1,000-W Xenon arc lamp served as the light source. Light was focused through a system of achromatic lenses and passed through a circular neutral-density wedge that provided linear attenuation of light energy. This wedge was used to equalize energy across conditions prior to spectral filtering.

Narrowband interference filters (20 nm half-bandpass) produced peak wavelengths of 420, 460, 500, 540, 580, 620, and 660 nm. A broadband white xenon condition was also included. Filtered light was projected onto a light shield with two small circular apertures (2 mm, ~ 4 arcmin), producing a homogeneous circular field (~ 6° diameter) on the shield. The apertures could be positioned adjacently (appearing as a single point) or separated gradually until participants perceived two distinct points. A collapsible baffle blocked light between the apertures during separation. A built-in digital micrometer recorded the separation threshold. Extensive baffling throughout the optical system minimized straylight.

An adjustable chin-and-forehead rest positioned the participant 67 in. (≈1.7 m) from the aperture shield, ensuring alignment with the optical axis.

### Calibration and energy control

Before testing, total radiant energy was calibrated for each wavelength. For each condition, the neutral-density wedge was adjusted to equalize energy at the aperture (628 μW). Energy was verified with a radiometer (UDT model S370 photodetector head positioned at the apertures). The maximum available energy occurred at 660 nm, and this value was used as the standard across all wavelengths.

Because the apertures were small and the stimulus plane was over 5 ft from the eye (~ 1.5 m), luminance at the retina was very low (below the detection range of a standard photometer; Extech Instruments, USA). Nevertheless, stimuli were clearly visible against the dark background. We note that stimuli were equated on a radiometric (energy) basis rather than a photometric (luminance) basis, meaning that brightness differences across wavelengths were not eliminated. Had stimuli been photometrically matched, substantially more short-wave energy would have been required, changing retinal illuminance and adaptation and thereby confounding wavelength-specific optical effects.

### Procedure

Testing was conducted in a darkened room. Participants were instructed using animated demonstrations of point separation and covered the nondominant eye with a patch. They rested their chin and forehead on the stabilized mount. To ensure alignment, a small fixation post with a pinhole along the optical axis was used before testing (removed prior to stimulus presentation).

**Fig. 1:**
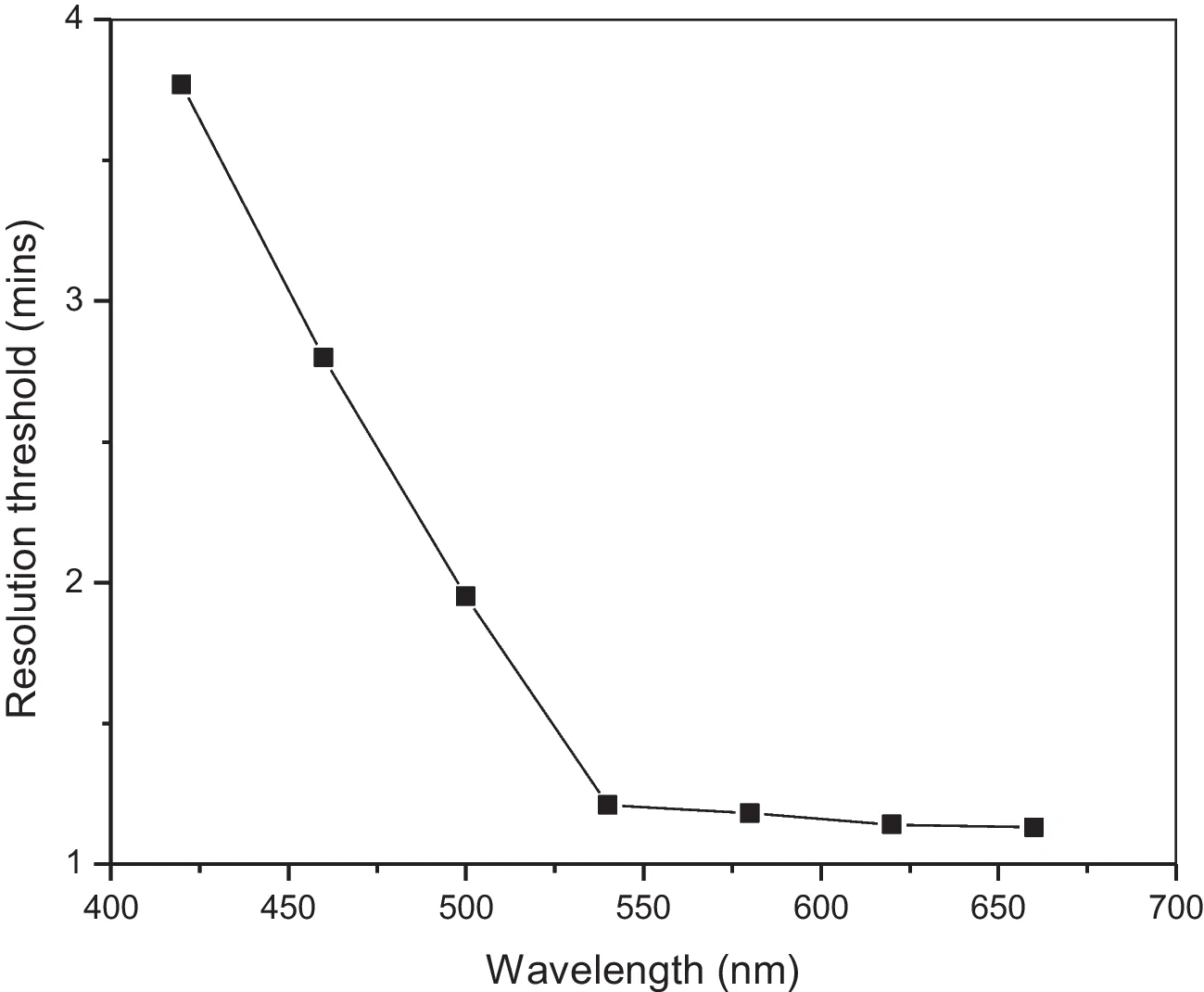
Two-point thresholds (arcmin) across wavelength (median values)

Each trial began with the micrometer at zero (apertures closed). Apertures were slowly separated until the subject reported perceiving two distinct points. The corresponding micrometer separation was recorded. The aperture was then closed and reset before the next trial. Each wavelength condition was tested three times in randomized order; mean values were used in the analyses. The procedure was based on an ascending method of adjustment. A descending series was not included in an effort to minimize testing time (due to the high number of conditions that we were testing). It is important to note, however, that ascending-only adjustment may slightly elevate threshold estimates (Ehrenstein & Ehrenstein, 1999), although we assume the effect was the same for each waveband and would not be expected to change the shape of our measured action spectrum (Fig. 1). Each experimental session lasted approximately 1 h.

### Control of optical variables

All participants were tested without refractive correction to avoid variability introduced by optical devices, but only those with VA > 20/40 unaided were included. Accommodation was not explicitly measured; however, the low luminance and scattered nature of the stimulus likely reduced effective chromatic accommodation. Testing distance (67 in.) introduced a minimal accommodative demand (~ 0.60 diopters).

Pupil size was not pharmacologically controlled, but testing was conducted under dim light in which pupil diameters are typically 3–6 mm in this age group (Stockman & Sharpe, 2006). Within this range, diffraction dominates at the lower end (Campbell & Gubisch, 1966) and higher-order aberrations at the upper end. Although individual differences in pupil size likely contributed to variability, they would not systematically bias the wavelength-dependent pattern, given the low luminance and within-subject design.

### Statistical analysis

All analyses were performed using SPSS, version 29. In this study, two hypotheses were tested: (1) that there would be wavelength-dependent differences in two-point thresholds, and (2) that iris pigmentation may also affect two-point thresholds. These hypotheses were non-directional in nature; consequently, both tails of the distribution were tested, with *p* < 0.05 as the criterion for statistical significance.

To test these hypotheses, we first tested assumptions for parametric tests such as normality, using the Shapiro–Wilk test, and homogeneity of variance, using Levene’s test. Across wavelengths, none of the thresholds were normally distributed (W = 0.813 at 420 nm; 0.743 at 460 nm; 0.648 at 500 nm; 0.679 at 540 nm; 0.525 at 580nm; 0.567 at 620 nm; 0.687 at 660 nm; and 0.459 for the broadband condition; *p* < 0.001 for all wavelengths), and all threshold variances were heterogenous (*F*(7,374.503) = 5.749, *p* < 0.001).

To isolate the effects of wavelength on two-point thresholds, the Friedman test was completed, and pairwise tests were Bonferroni corrected for multiple tests. To determine the effects of iris color on two-point thresholds between and across wavelengths, we used a general linear mixed-effects model with wavelength as a within-subject factor, iris color and lightness as between-subjects predictors, and subject as a random effect. Because threshold values were non-normally distributed and variances were heterogeneous, models were estimated using REML with Satterthwaite degrees of freedom. Bonferroni-corrected post hoc tests were then completed.

## Results

Descriptive statistics for the wavelength variables are provided in Table 2. As shown in the table, the wavelengths each displayed a large range with the largest separation at the shortest wavelengths.

**Table 2:** Descriptive statistics of the two-point separation thresholds specified by peak wavelength (values expressed in arc minutes of visual angle)

Wavelengths (nm)	Median	Range
420	3.77	0.67–15.63
460	2.80	0.48–17.45
500	1.95	0.34–15.36
540	1.21	0.29–8.22
580	1.18	0.30–8.09
620	1.14	0.36–11.10
660	1.13	0.43–8.05
Broadband	0.69	0.20–11.6

### Spatial resolution across the wavelengths

Two-point thresholds were recorded as the distance in mm needed for a participant to detect two separate points of light separating from a single point in space. That distance was converted to visual angle, and visual angle at threshold was then analyzed. There was a significant difference in two-point thresholds by wavelength (χ ^2^(7, *N* = 60) = 243.84, *p* < 0.001). Post hoc pair-wise comparisons between individual wavelengths, Bonferroni corrected for multiple tests, are presented in Table 3. Broadband light always yielded smaller two-point thresholds (i.e., less separation of the points was necessary for participants to perceive two distinct points) than individual wavelengths (adjusted *p* ≤ 0.009 for all comparisons). Longer wave thresholds (660 and 620 nm) were significantly different from shorter-wave (500, 460, and 420 nm; adjusted *p* ≤ 0.007 for all comparisons), but were not significantly different from each other, or from mid-wave thresholds. Mid-wave thresholds (580 and 540 nm) were not significantly different from each other or from long-wave thresholds but were significantly different from shorter-wave thresholds (460 and 420 nm; adjusted *p* = 0.000), and 580 nm was also significantly different from 500 nm (adjusted *p* = 0.001). Thresholds at 500 nm were not significantly different from 460 nm but were different from 420 nm (adjusted *p* = 0.000). Thresholds at 460 nm were not significantly different from 420 nm. For all conditions except the broadband condition, the longer the wavelength, the smaller the visual angle subtended by the gap between the two points at threshold. In other words, for longer wavelengths, thresholds were smaller, indicating that participants needed less distance between the two points to discriminate two separate points. A graphical representation of these results is provided in Fig. 1.

**Table 3:**
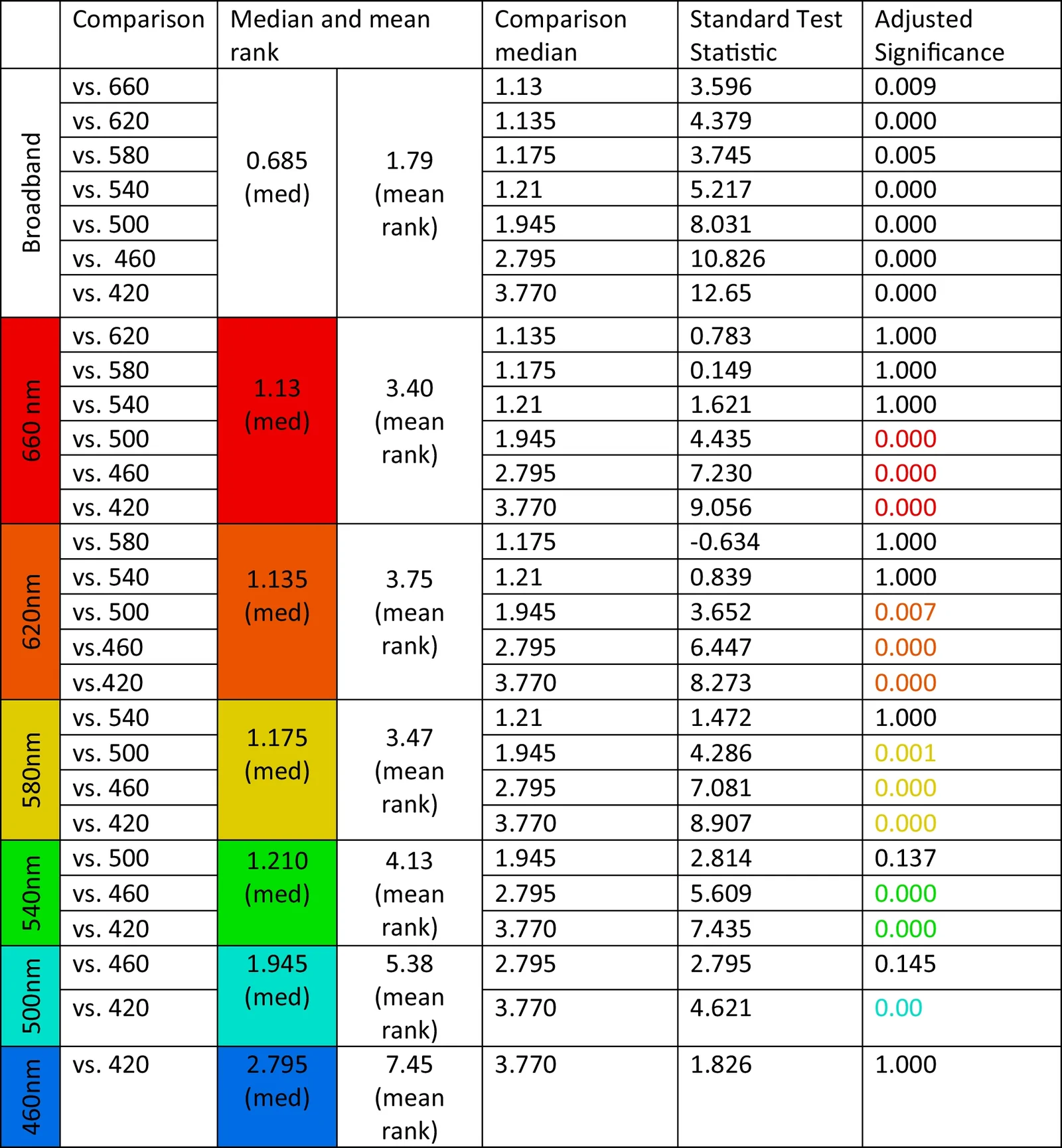
Comparisons between two-point thresholds (arcmins) at the tested wavelengths, Bonferroni corrected for multiple comparisons

### Effect of iris pigmentation on spatial resolutions

There was a significant effect of iris color (*F*(3, 47.587) = 6.953, *p* < 0.001), a significant effect of iris lightness (*F*(2, 47.587) = 8.256, *p* < 0.001), and a significant combinatory effect of color and lightness (*F*(5, 47.587) = 4.196, *p* = 0.003) on thresholds.

#### Iris lightness

Estimated marginal means indicated that thresholds were highest for lighter irides (*M* = 3.992, *SE* = 0.356), compared to medium irides (*M* = 2.394, *SE* = 0.251), or dark irides (*M* = 2.068, *SE* = 0.336). Post hoc comparisons (Bonferroni adjusted) showed that these differences were significant for light versus medium irides (*p* < 0.001) and light versus dark irides (*p* < 0.001), but not for medium versus dark irides (*p* = 0.917; Table 4).

**Table 4:** Estimated marginal means and standard errors for color and iris combinations

Iris lightness	Iris color*	Estimated marginal mean	Standard error
Light	Blue	9.888	1.282
	Green	3.620	0.643
	Hazel	2.074	1.282
	Brown	1.802	1.282
Medium	Blue	2.839	0.525
	Green	1.898	0.907
	Hazel	1.630	0.525
	Brown	2.463	0.373
Dark	Blue	1.539	0.907
	Green	1.760	1.282
	Brown	1.875	0.266

#### Iris color

Estimated marginal means indicated that thresholds were highest for blue irides (*M* = 4.373, *SE* = 0.345), compared to green irides (*M* = 2.832, *SE* = 0.351), hazel irides (*M* = 1.992, *SE* = 0.407), and brown irides (*M* = 2.052, *SE* = 0.304). Bonferroni-adjusted post hoc comparisons showed that the differences between blue irides and green irides (*p* = 0.003), hazel irides (*p* < 0.001), and brown irides (*p* < 0.001) were significant. There were no significant differences between green, hazel, and brown irides (see Table 4).

#### Iris color + lightness

Estimated marginal means and standard errors for color-lightness combinations are presented in Table 4. Participants with light blue irides (*M* = 9.888, *SE* = 1.282) demonstrated significantly higher thresholds than light green irides (*M* = 3.620, *SE* = 0.643; adjusted *p* = 0.029), hazel irides (*M* = 2.074, *SE* = 1.282; adjusted *p* = 0.012), and brown irides (*M* = 1.802, *SE* = 1.282; adjusted *p* = 0.003). There were no significant differences between other colors within the light iris category, or between any other color-lightness combination.

## Discussion

This study examined how wavelength influences fine spatial resolution under equal-energy conditions using a two-point discrimination task. The main finding was a robust wavelength dependence: resolution thresholds were elevated for short-wave stimuli and lower for longer wavelengths. Importantly, this pattern cannot be attributed to a single factor but rather likely reflects the combined influence of both optical and, possibly, neural factors.

While a Rayleigh-type function (λ⁻^4^) closely approximated the observed pattern (r^2^ = 0.97), this does not imply that classical Rayleigh scatter alone is the mechanism underlying the effect. In fact, unlike the atmosphere, the ocular media are composed of large molecules and organized proteins that do not readily produce classic Rayleigh scattering (Boettner & Wolter, 1962). The shape of the longitudinal chromatic aberration (LCA; r^2^ = 0.96) function also closely matched our empirical data. Short-wave light is particularly subject to LCA, and while accommodation can partially compensate for narrowband stimuli (Fernandez-Alonso et al., 2024; Rynders et al., 1998), the scattered light in our setup may have limited effective chromatic accommodation so LCA could also be a contributor. There are a number of optical phenomena that likely, collectively, explain these results. Alternative mechanisms such as Tyndall scatter from colloidal particles (e.g., crystallin proteins in the lens) could, for instance, contribute (Delaye & Tardieu, 1983). What is clear is that numerous wavelength-dependent processes, such as chromatic aberration and wavelength-weighted straylight, occur concurrently. Without direct wavefront or straylight measurements, the present behavioral data cannot uniquely partition the relative contributions of chromatic aberration, scatter, and neural sampling. Nonetheless, the strong short-wavelength elevation in thresholds, combined with moderation by irides pigmentation, supports a predominantly optical origin rather than a post-receptoral mechanism alone.

Individual variation further highlights the role of multiple interacting factors. Resolution was moderated by iris pigmentation (largely blue/light irides), with darker irises associated with lower thresholds, consistent with reports that darker pigmentation reduces intraocular scatter and enhances image quality (Coppens et al., 2006). Iris pigment density and macular pigment optical density (MPOD) are known to co-vary, with darker irides associated with higher MPOD independent of dietary carotenoid intake (Hammond et al., 1996). Both melanin in the iris and carotenoids in the macula reduce short-wave retinal irradiance and intraocular straylight, mitigating disability glare and enhancing fine spatial resolution in central vision (Buabeng & Hammond, 2025). Pupil size, which modulates diffraction at small diameters and aberrations at large diameters (Campbell & Gubisch, 1966), may have contributed to between-subject variability. Although these variables may explain individual differences, they would likely not alter the overall wavelength-dependent function given the comparable adaptation state across conditions.

Our results align with a broader principle: action spectra are pervasive in vision. Many visual functions, from spectral sensitivity (CIE, 1932) to glare discomfort (Stringham et al., 2003), photostress recovery (Ham et al., 1976), and photophobia (Stringham et al., 2011), show disproportionate short-wave effects. The present findings extend this principle to fine spatial resolution, reinforcing that short-wave light can potentially degrade performance even under low-energy conditions.

Several limitations must be emphasized. First, the equal-energy approach isolates wavelength effects but does not equate perceived luminance, which may confound interpretation relative to natural viewing. Second, although we excluded participants with significant refractive error, and pupil size likely did not vary significantly in these younger participants under our controlled luminance conditions, it is still the case that higher-order aberrations and subtle differences in accommodation could have influenced the results. We also used only an ascending method of adjustment which could have resulted in systematically higher thresholds (although the general shape of the curve was likely less affected). Finally, these findings are specific to the resolution task at low luminance; other tasks driven more strongly by scatter (e.g., glare disability) may produce different spectral dependencies.

In summary, spatial resolution thresholds show systematic wavelength dependence, likely arising from the combined contributions of scatter, chromatic aberration, diffraction, and neural sampling limitations. These results underscore the importance of considering wavelength as a determinant of visual performance and suggest that ocular pigmentation may play a protective role in mitigating scatter-related losses.

## Data Availability

The datasets generated during the current study are available from the corresponding author on reasonable request.

## Declarations

### Data accessibility

The data that support the findings of this study are available upon reasonable request from the authors.

### Open practices statement

None of the data or materials for the experiments reported here is available, and none of the experiments was preregistered.
